# Comment on “Economic Burden and Medical Insurance Impact of the Different Dialysis for End-Stage Renal Diseases”

**Published:** 2019-04

**Authors:** Mohsen Bayati

**Affiliations:** Health Human Resources Research Center, School of Management & Information Sciences, Shiraz University of Medical Sciences, Shiraz, Iran

## Dear Editor-in-Chief

A recently-published paper by Gao et al. (1), studied the cost of two popular interventions for End Stage Renal Disease including hemodialysis and peritoneal dialysis in a hospital in China. It also compared the hemodialysis and peritoneal dialysis cost between two main medical insurance organizations: New Cooperative Medical Scheme and the Urban Employees’ Medical Insurance. As the authors mentioned in the title and several times in the manuscript, the primary aim of their study was to estimate the economic burden of hemodialysis and peritoneal dialysis. However, we expect more comprehensive analysis to assess economic burden of diseases. In this regard, I have some comments.

First, in all types of economic evaluation the first step is determining perspective or viewpoint of study. In related researches on economic burden of hemodyalisis and peritoneal dialysis, the perspective have been clearly specified (2–4). Moreover, it would be perfect if the societal perspective be chosen for economic burden of diseases study. Rizk et al. estimated the cost of hemodialysis in Lebanon from the society viewpoints (2).

Second, in a precise estimation of economic burden of hemodialysis and peritoneal dialysis, we should cover all costs burdened on population or health system. In this regard, the structure of cost of illness study usually is used. In cost of illness, total healthcare cost is categorized in three types: direct, indirect, and intangible cost (5). In practice, because of methodological problems, the intangible cost not considered. However any perfect economic burden or cost of illness analysis should assess the direct and indirect costs. In a study, the direct medical cost; direct non-medical cost such as transportation and food cost; and indirect cost (productivity loss) such as absenteeism and quit work of hemodialysis were included (3). Rizk et al. also covered all direct and indirect costs of hemodialysis in Lebanon in different classification including healthcare sector cost (hemodialysis, health care professionals, medications, hospitalization, professional home care), costs to patient and family (informal care and productivity losses), and costs in other sectors (travel) (2). However in the Gao et al study (1), only direct medical cost of hemodialysis and peritoneal dialysis was considered.

An important issue is that, according to literature, share of direct non-medical and indirect cost of total cost of hemodialysis is noticeable. For example in Jordan study, share of direct non-medical (11%) and indirect costs (48%) accounts for about 59% of total cost of hemodialysis. In Lebanon, items such as travel (4.09), productivity losses (2.06) and informal care (2.12) were about 10% of total cost of hemodialysis.

As conclusion, the main result of Gao et al. study (1), the cost of hemodialysis is higher than peritoneal dialysis, is in light with other studies (4, 6). However, because of mentioned issues, the magnitude of total cost or economic burden of these two dialysis methods is in doubt.
